# Improving the efficiency of RMSProp optimizer by utilizing Nestrove in deep learning

**DOI:** 10.1038/s41598-023-35663-x

**Published:** 2023-05-31

**Authors:** Reham Elshamy, Osama Abu-Elnasr, Mohamed Elhoseny, Samir Elmougy

**Affiliations:** Faculty of Computers and Information, Mansoura University, Mansoura, Egypt

**Keywords:** Mathematics and computing, Computer science

## Abstract

There are several methods that have been discovered to improve the performance of Deep Learning (DL). Many of these methods reached the best performance of their models by tuning several parameters such as Transfer Learning, Data augmentation, Dropout, and Batch Normalization, while other selects the best optimizer and the best architecture for their model. This paper is mainly concerned with the optimization algorithms in DL. It proposes a modified version of Root Mean Squared Propagation (RMSProp) algorithm, called NRMSProp, to improve the speed of convergence, and to find the minimum of the loss function quicker than the original RMSProp optimizer. Moreover, NRMSProp takes the original algorithm, RMSProp, a step further by using the advantages of Nesterov Accelerated Gradient (NAG). It also takes in consideration the direction of the gradient at the next step, with respect to the history of the previous gradients, and adapts the value of the learning rate. As a result, this modification helps NRMSProp to convergence quicker than the original RMSProp, without any increase in the complexity of the RMSProp. In this work, many experiments had been conducted to evaluate the performance of NRMSProp with performing several tests with deep Convolution Neural Networks (CNNs) using different datasets on RMSProp, Adam, and NRMSProp optimizers. The experimental results showed that NRMSProp has achieved effective performance, and accuracy up to 0.97 in most cases, in comparison to RMSProp and Adam optimizers, without any increase in the complexity of the algorithm and with fine amount of memory and time.

## Introduction

When deciding to build a structure in Deep Learning (DL)^1^ to solve a specific problem, many questions arise for creating a powerful structure with great accuracy, and optimal training time. The most popular questions are: which hyper parameters, such as learning rate^2^, batch size^3^, momentum^4^, and weight decay^5^, should be used? How to tune them to achieve the desired result? Which optimizer is the optimal one to answer the research problem? Actually, there are no specific guidelines for setting up an optimal DL structure with these parameters suitable for solving all problems or all data sets. Recently, there have been many researches interested in investigating hyper parameters and finding out the suitable values of these parameters for solving different problems. However, this paper focuses on the work mechanism of optimizers, and how to enhance their performance with regard to solving specific problems.

The aim of any optimizer is to minimize the loss function^6^, which is the difference between the actual output from the structure, and the desired output. There are many types of optimizers that can be used to achieve the minimum value for the loss function. Many papers have proposed new enhancement techniques on the traditional optimizers such as Stochastic Gradient Descen (SGD)^7^, AdaGrad^8^, AdaDelta^9^, Nadam^10^, Adam^11^, and RmsProp^12^. They include modification of many aspects such as the momentum, and learning rate. This paper proposed an algorithm, NRMSProp, to improve the performance of RmsProp optimizer, by adding a further step that involves calculating the Nestrove for a further point, with respect to the average of the past squared gradients for the current point. The performance of NRMSProp model is compared with the performance of the RMSProp and Adam optimizers under the same conditions.

The remainder of this paper is divided into five sections. The second section contains the “Literature review”. Section "Proposed Model NRMSProp" discusses the proposed optimizer in detail. Section "Experiments" presents the experiments on different datasets. Finally, section “Conclusion” concludes the work.

## Literature review

### Background

In this subsection, the focus is on the idea of how the optimizers work in Deep Neural Networks (DNNs). It presents some previous enhancement techniques of different traditional optimizers that deal with DL problems. Then, some modified versions of these optimizers are presented, with discussing how they work on different problems under different conditions. Training deep models is effectively remaining one of the most required tasks for researchers and practitioners in both real-world DL research, and application work. So far, the vast majority of the deep model training is based on the back propagation algorithm, which propagates the errors from the output layer backward, and uses gradient descent-based optimization algorithms to update the parameters layer by layer. Therefore, in order to achieve an effective model, the suitable optimizer to handle the problem should be chosen. There are different types of optimizers, such as Gradient Descent based Learning (GD), Momentum based Learning, Adaptive Gradient based Learning, and Momentum Adaptive Gradient based Learning Algorithms.

#### Gradient descent based learning (GD) algorithms

GD^13^ is utilized to reduce some functions by iteratively moving in the direction of steepest descent as defined by the negative of the gradient. It is used to update the parameters’ model in DL. In addition; there are multiple types of GD, such as the stochastic gradient batch (vanilla)^14^, gradient descent, or mini-batch gradient descent. The main difference between Batch Gradient Descent (BGD)^15^ and Stochastic Gradient Descent (SGD)^16^ is the cost of one example for each procedure in SGD is only computed. In contrast, in BGD, the cost for all training examples in the dataset has to be computed. This extremely quickens the neural networks. Basically, this is what stimulates utilizing SGD. SGD is utilized to update parameters in a DL, as shown in Eq. (1). Besides, this equation is employed to update parameters in a backwards pass, with the help of back propagation^17^, to compute the gradient. Each parameter, *θ*, is taken and updated by getting the original parameter and subtracting the learning rate times the ratio of change.

1 $$\theta = \theta - \eta \cdot \nabla_{F} \;(\theta )$$

It is noteworthy that in order to solve these drawbacks of SGD, an enhancement should be done. Mini Batch Gradient Descent^18^ would be adopted because it has the best of the two approaches. It also executes an update for each batch of n training examples in every batch. The following sub-section is devoted for discussing the other types of optimization algorithms.

#### Momentum based learning algorithms

Instead of depending only on the gradient of the current step to steer the search, momentum, the gradient of the previous steps is considers^19^. The gradient descent equations are changed as given in Eq. (2).

2 $$\mu (\tau ) = \Upsilon \mu (\tau - 1) + \eta \nabla_{F} (\theta )$$

**Figure 1:**
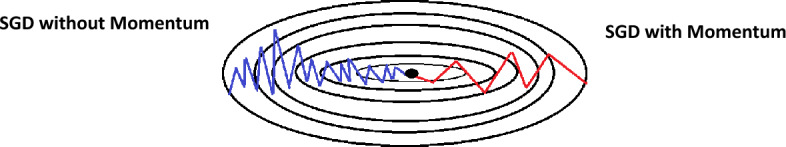
SGD without momentum via with momentum.

In the end, the parameters are updated through θτ + 1 = θτ − µτ. Thus, this allows us to fine-tune the updates to the slopes of our error function, which speeds up SGD, as shown in Figure 1 which illustrates how Momentum speeds up the SGD in training process^20^. Moreover, it helps adapt the updates to every individual parameter to execute larger or smaller updates relying on their impact. In the sub-section below, some adaptive algorithms are introduced.

**Figure 2:**
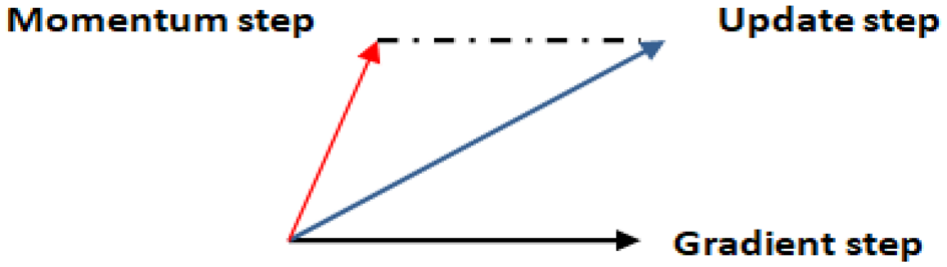
The effect of Nestrove on the Gradient step.

The Nesterov Momentum update^21^ is a significantly modified adaptation of the momentum update that has recently gained popularity. It has higher efficiency convergence promises for convex functions. It also performs slightly better in practice than the ordinary momentum. When the current parameter vector is at x, it is interfered from the momentum update above that the momentum term alone is about to nudge the parameter vector by µu*υ. Therefore, to compute the gradient, the future approximate position, *x* + µ u*υ, should be considered as a looked-ahead^22^ at a further point to stop at. As a result, instead of computing the gradient at the previous position, *x*, compute it at *x *+ µu*υ as clarified in Fig. 2.

#### Adaptive gradient based learning algorithms

This subsection investigates a group of learning algorithms, such as Adagrad, Adadelta, and RMSProp,that use adaptive learning rates to update variables. Adagrad^23^ is a technique that permits the learning rate to adapt depending on the parameters. Thus, it provides big updates for infrequent parameters, and small updates for frequent parameters. Also, it is well known for being appropriate for handling sparse data. This technique utilizes a different learning rate for each parameter at a time step based on the past gradients that were calculated for that parameter. Adadelta^24^ is considered to be an expansion of AdaGrad that lean towards to eliminate the decay caused by the learning rate. This strategy limits the window of collected past gradients to few settled estimate weights rather than pressing the entire squared first-order derivative by using a decay average. RMSProp can be thought of as an extension of AdaGrad which uses a moving average (MA) of the partial derivatives instead of the sum in the calculation of the η for each instance as clarified in Eqs. (3) and (4) where S is the squared of decay average.

3 $$S_{t } = \beta_{1 } S_{t - 1} + (1 - \beta_{1} )g^{2}$$

4 $$\theta_{t + 1} = \theta_{t} - \frac{\eta }{{\sqrt {s_{t} + \epsilon } }} {\text{g}}_{{\text{t}}}$$

It is available to compute learning rates for every parameter. Moreover, individual changes for every parameter could be calculated and packed independently.

#### Momentum and adaptive gradient based learning algorithms

This subsection discusses some learning algorithms that incorporate the benefits of the previous two algorithms such as Adaptive Moment Estimation (Adam)^25^ which can be defined as another approach which calculates the adaptive learning rates for every parameter. Besides, it keeps the average of all past squared gradients, such as AdaDelta. Adam is one of the effective optimization approaches in adaptive algorithms. It is an SGD algorithm that relies on the concept of momentum to quickly and effectively arrive at the loss function's global minimum. This aids in efficiently modifying the learning rate for each parameter, hastening the time to convergence to the minimum. Adam calculates distinct adaptive learning rates based on the first and second moment values for various parameters as clarified in Eqs. (5) and (6).

5 $$\hat{\mu }_{{\text{t}}} = \frac{{\mu_{{\text{t}}} }}{{1 - \beta_{{\text{t}}}^{1} }}$$

6 $${\hat{\text{v}}}_{{\text{t}}} = \frac{{{\text{v}}_{{\text{t}}} }}{{1 - \beta_{{\text{t}}}^{2} }}$$

where µ is the mean and υ is the variance of the first- order derivative in the same order. Equation (7) gives the final step of updating θ.

7 $$\theta_{{{\text{t}} + 1}} = \theta_{{\text{t}}} - \frac{\eta }{{\sqrt {{\hat{{v}}}_{{\text{t}}} + \epsilon } }} \hat{\mu }_{{\text{t}}}$$

Adam performs well in practice, and provides results that can be favorably compared to other optimization algorithms since it achieves the optima fast, and its performance is quiet quick and so effective. The technique of adaptive algorithms has the ability to also correct and fix each issue that can be confronted in other optimization algorithms that may cause fluctuation in the loss function adaptive techniques. There is an issue concerning the learning rate to be "just right", which is so tricky. If it is selected too small, there will be no progress. On the other hand, if it is too large, the solution will fluctuate, and be in the worst condition; it may even diverge. So, if is specified and selected automatically, or even this step is avoided, the second order techniques, which search not only for the value and gradient of the objective but also for its curvature, can be beneficial in this case.

### Related work

Dozat^26^ improved Adam and explained and pointed out how to regenerate of Nesterov Accelerated Gradient (NAG) to be more direct and precise concerning performance. In this work, he had not executed the step of modifying the parameters with just the momentum procedure to compute the gradient in order to get back to the main parameter state. After that, the momentum procedure continues again during the real authentic update. Furthermore, the time of the momentum procedure can be applied only one time during the update of the previous time phase. Tato and Nkambou^27^ introduced additional hyperparameters to Adam optimizer that preserves the direction of the gradient through ingrained optimization execution. On the other hand, Keskar and Socher^28^ created a modified version of Adam (AAdam) to accelerate and quicken its performance. They aim to obtain a better minimum for the loss function, in comparison with the main algorithm, by extracting some thoughts from the momentum relying on the optimizer and exponential decay methodology. The clarified also that the procedure magnitude is produced by Adam to adjust the parameters. It is the way that the new procedure takes into account both of the direction of the gradient and the modification applied to the previous procedures. The authors also used MNIST data set for evaluation. The results showed that AAdam achieves the best results particularly on the validation set, even in the cases that require more memory. Also, they showed that it surpasses and outturns Adam and NAdam in decreasing training, and validation loss, and achieve better accuracy than the other methodologies.

Reddi et al.^29^ introduced a combined hybrid methodology that begins with a flexible technique and then switches to SGD when convenient. They also presented SWATS which is considered as a simple methodology that converts from Adam to SGD, when a triggering case is satisfied. The case which is introduced is related to the projection of the Adam procedures on the gradient subspace. The cost of the observation procedure of this case is very low, and does not expand the number of hyperparameters in the optimizer. Furthermore, the authors created both switchover point and learning rate for SGD after the switch is assigned as a part of the algorithm. Due to these requirements, no convenient effort is added. Additionally, the authors demonstrated the adequacy of this methodology on ImageNet data sets. The results clarified that the proposed methodology compared with to SGD, while retaining the beneficial qualities of Adam such as the insensitivity, and quick initial advance of the hyperparameter. Hoseini et al.^30^ proposed an algorithm to enable the training model in DCNN to switch between RMSprop and Nestrove optimizers to grantee that the AdaptAhead optimizer does not make any change of the structure of RMSprop and Nestrove algorithms. AdaptAhead built three switches; the first is responsible for a set the value of hyper-parameter norms, which corresponds to norm-1, Euclidean norm, and max-norm. The second switch determines whether gradients whether in the normal or in the Nesterov method. The third switch determines when the learning rate works whether by applying the calculated norm in an adaptive manner, or in the normal manner based on Nesterov method. Xue et al.^31^ suggested an approach to enhance the training of feed-forward NNs, which integrates the advantages of Differential Evolution and Adam. This approach explores the search space using a population-based method and adaptively modifies the learning rate to hasten convergence. Their results show that this proposed approach exhibited impressive outcomes in terms of accuracy and convergence speed across.

Wang et al.^32^ presented architecture for communication-efficient compressed federated adaptive gradient optimization, FedCAMS, which tackles the adaptively problem in federated optimization techniques while substantially reducing communication overhead. He suggested a universal adaptive federated optimization framework, FedAMS, as a base for FedCAMS. FedAMS, that includes different iterations of Adam characteristic max stabilization techniques. They offer an enhanced theoretical examination of the convergence of adaptive federated optimization, based on which they demonstrate that their suggested FedCAMS accomplishes the same convergence rate as its uncompressed counterpart FedAMS with a number of magnitude less communication cost in the no convex stochastic optimization context.

## Proposed model NRMSProp

The main contribution of this work is to propose a model to enhance the performance of Adaptive Gradient based Learning algorithms in different ways such as the time and the accuracy. The proposed NRMSprop model gains its power from the advantages of Nestrove approach and the way in which RMSprop is calculating the gradients. The steps of NRMSprop are shown in Algorithm 1.

**Table:** 

Algorithm 1. NRMSProp			
η: the learning rate β1, β2: smoothing parameters f(θ): the objective function with parameter θ			
While: f(θ) does not converged do			
Step 1:	τ = τ + 1		
Step 2:	compute the gradient at step τ	$g_{t} = \nabla f(\theta_{t - 1} )$	(8)
Step 3:	calculate Nestrove momentum vector	$\mu_{t } = \beta_{2}^{t} \mu_{t - 1} + (1 - \beta_{2}^{t} )g_{t}$	(9)
Step 4:	calculate the square of exponential moving average with term (g_t_ − µ_t_)	$s_{t } = \beta_{1}^{t} s_{t - 1} + (1 - \beta_{1}^{t} )(g_{t} - \mu_{t} )^{2}$	(10)
Step 5:	correction of Bias	$\hat{\mu }_{t} = \frac{{\mu_{t} }}{{1 - \beta_{t}^{2} }}$	(11)
Step 6:	Apply the update of θ	$\theta_{t + 1} = \theta_{t} - \frac{\eta }{{\sqrt {s_{t} + \epsilon } }} \hat{\mu }$	(12)
End While			

As clarified in Algorithm 1, Step 1 allocates the initial time, *τ*, and Step 2 calculates the initial gradient *g*_*t*_ which is the main parameter that optimizers of DL used to update the overall weights and the other parameters of the optimization process as the end of the process in Step 6. In the first iteration, *g*_*t*_, is computed depending on initial random point as a start point. As the goal of optimization process is reducing the difference between this start point and the actual target minima NRMSprop computes the Nestrove vector *µ* in Step 3 and adding $\beta_{2}^{t}$ to its computations that can effectively affect on how to apply look-ahead technique by calculating the gradients not at the current point, but with respect to the approximate future point. In Step 4, NRMSprop keeps the value of all past gradients as a history parameter of each movement and calculates the exponential moving average (s). Keeping this history helps NRMSprop to be more stable and prevents overshoots when the target minima is very close. As in adaptive learning technique, the step size is not a static value for all iterations, NRmsprop takes care of the step size and adapts it for the optimal value depending on the distance between the current position and the minima, so there is a need to penalty the gradient with µ in term ${\text{g}}_{{\text{t}}} - \mu_{{\text{t}}}$ to reduce the step size if the target point is near to the current position. To decide the next step and choose the right direction, NRMSprop calculates the correction of Bias $\hat{\mu }_{{\text{t}}}$ in step 5. Finally in step 6 we update the weights θ by the form of Eq. (12) with adding Nestrove by replacing ($\hat{\mu }_{{{\text{t}} - 1}}$) of the earlier step by ($\hat{\mu }_{{\text{t}}}$) of the current momentum vector to get $\theta_{{{\text{t}} + 1}}$ as the last step of NRMSprop. To examine this proposed method we did experiments on Fashion-MNIST, CIFAR-10 and Tiny- ImageNet datasets using three Adam, RMSProp and the proposed optimizer NRMSPROP under same environment to compare the result of NRMSProp with the others optimizers, the next subsection show the experiments.

## Experiments

### Datasets description

**Figure 3:**
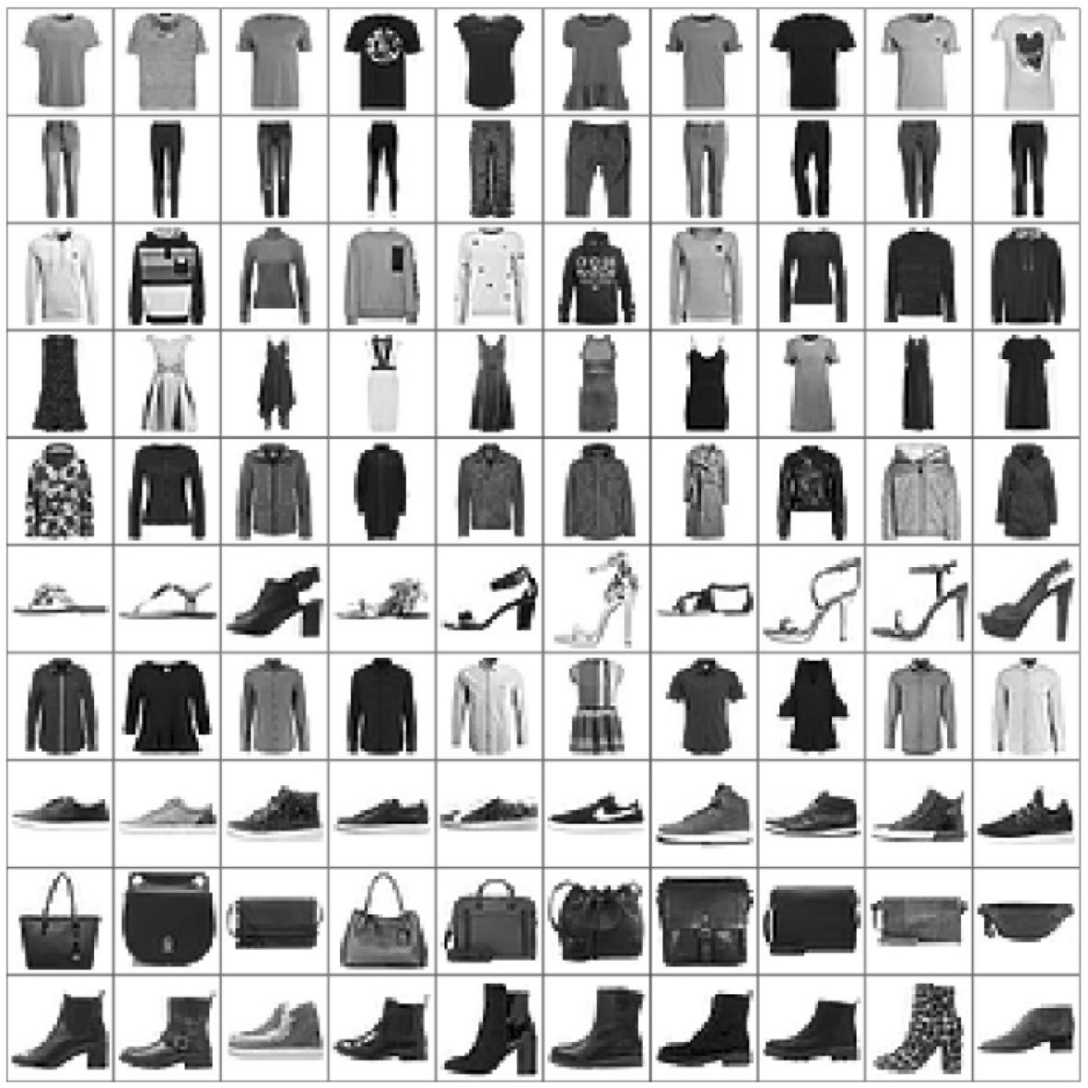
Class names and example images in Fashion-MNIST dataset^34^.

**Figure 4:**
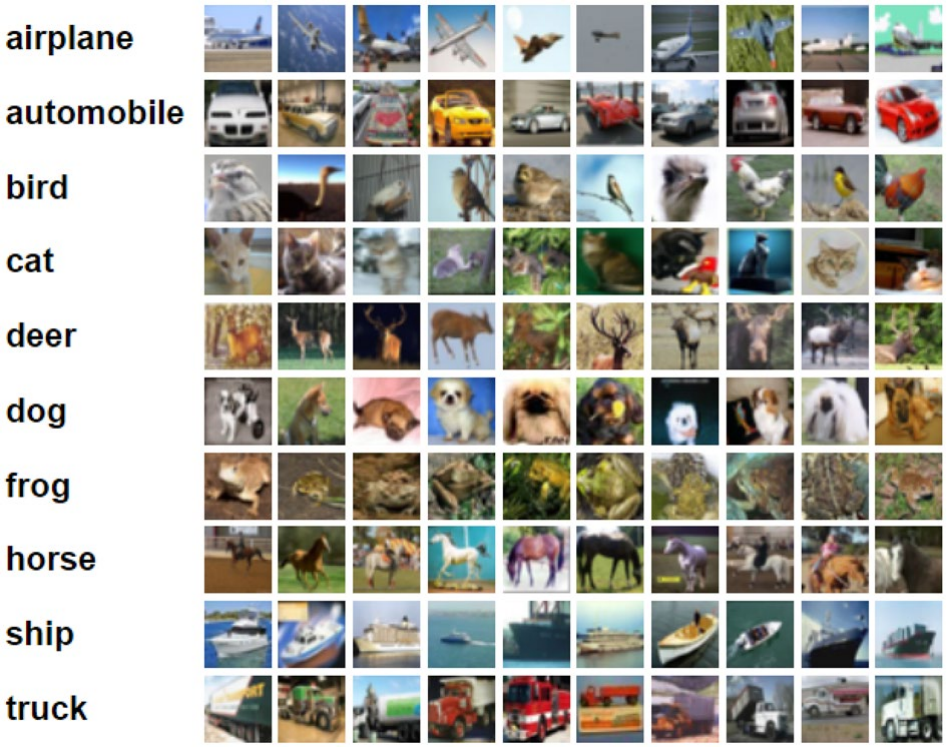
Class names and example images in CIFAR-10 dataset^35^.

**Figure 5:**
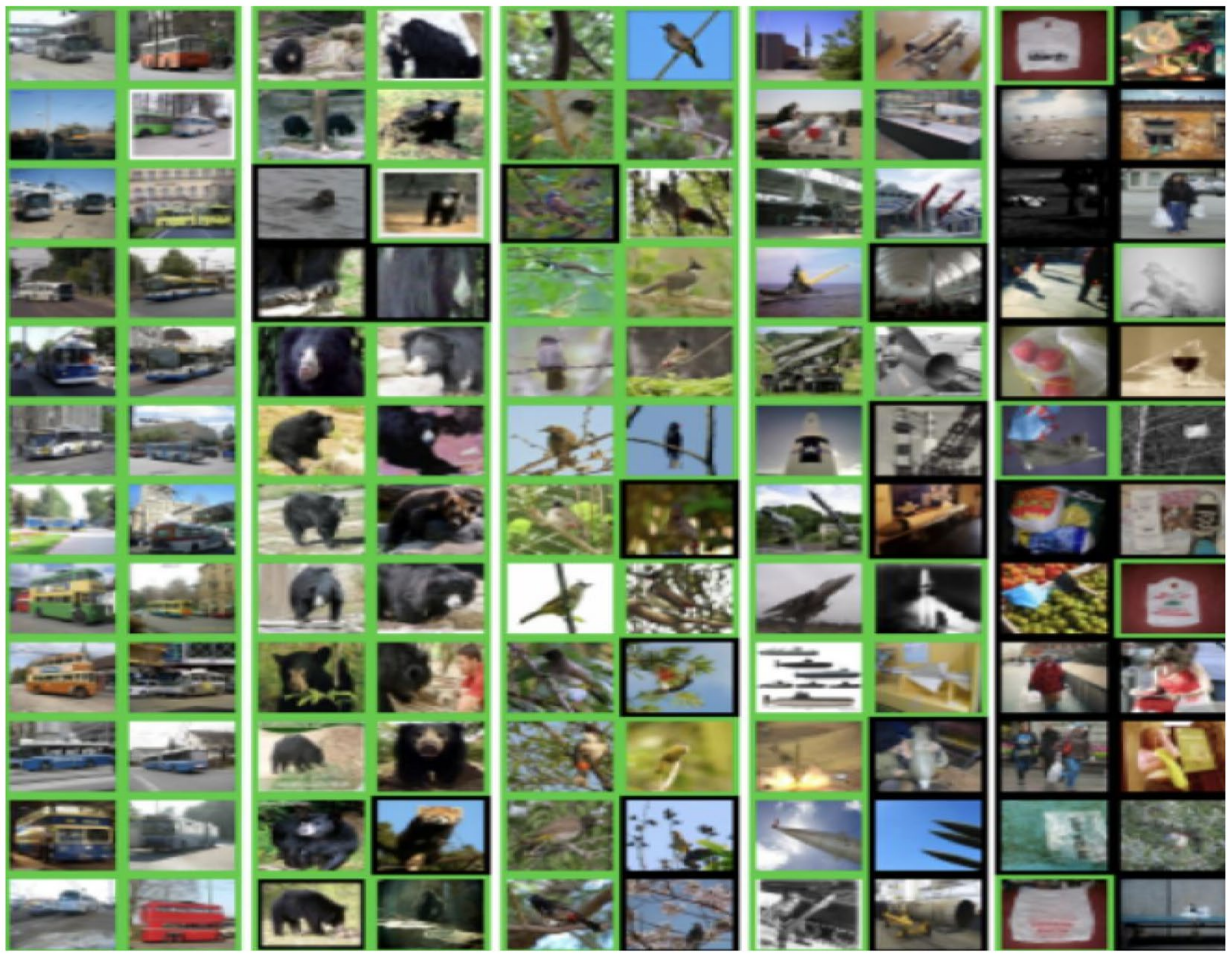
Sample of images in Tiny ImageNet dataset^37^.

Fashion-MNIST^33^ consists of 60,000 28 × 28 grayscale images of 10 fashion categories, along with a test set of 10,000 images. Figure 3 shows these 10 categories^34^. CIFAR-10 data^35^ includes 6000 images per class in 10 classes totaling 60,000 32 × 32 colour images; sample of this dataset are shown in Fig. 4. These images are splitting into 10,000 test photos and 50,000 training images are available. Tiny ImageNet dataset^36^ is a version of Image Net dataset. It is a container of 200 categories in which there are 100,000 images in these categories and 10,000 images for each validation and test processes. Sample of this dataset are shown in Fig. 5.

Accuracy (ACC), Precision (PREC), Recall (REC), and F1-score (F1) criteria, presented in Eqs. (13), (14), and (15), are used to evaluate NRMSProp, and to compare it with Adam and the original RmsProp optimizers^38^.

13 $${\text{ACC}} = \frac{{{\text{TP}} + {\text{TN}}}}{{{\text{TP}} + {\text{TN}} + {\text{FP}} + {\text{FN}}}}$$

14 $${\text{PREC}} = \frac{{{\text{TP}}}}{{{\text{TP}} + {\text{FP}}}}$$

15 $${\text{REC}} = \frac{{{\text{TP}}}}{{{\text{TP}} + {\text{FN}}}}$$

16 $${\text{F}}1 = \frac{{2 {\text{TP}}}}{{2{\text{ TP}} + {\text{FP}} + {\text{FN}}}}$$

### The model structure

**Figure 6:**
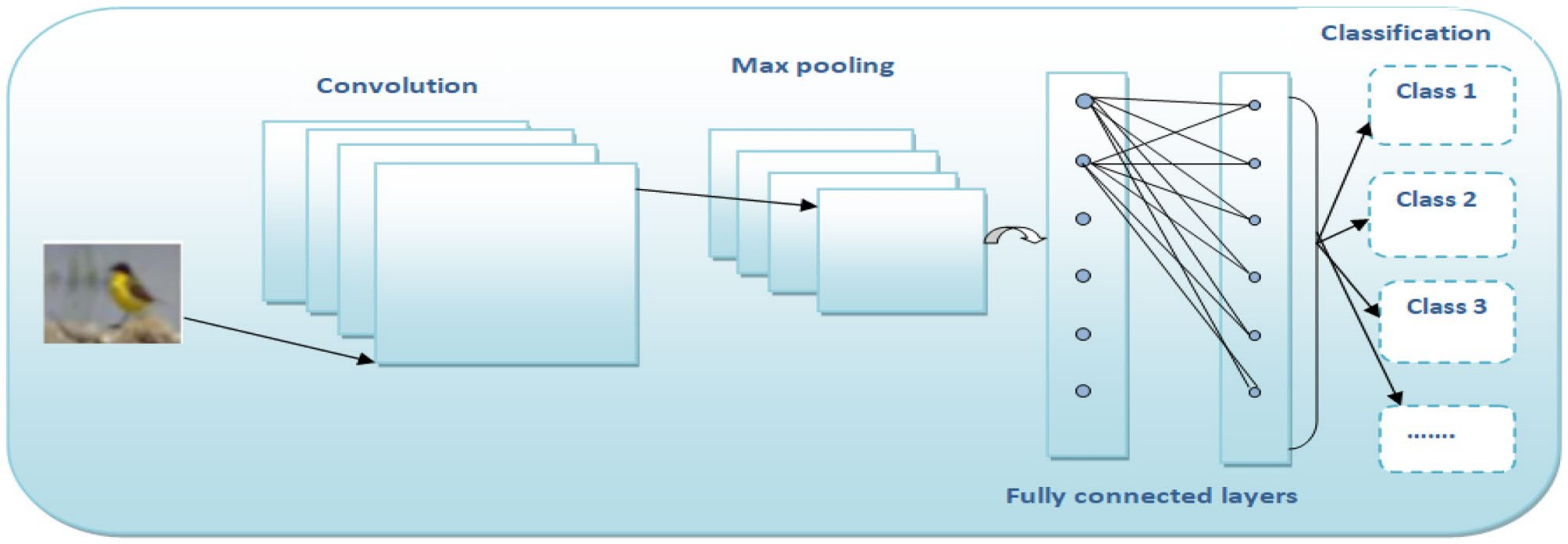
CNN layers.

To examine NRMSProp, we used two models: a simple Convolution Neural Network (CNN)^39^ model and ResNet model. Figure 6 illustrates the structure of CNN layers^40,41^.

Table 1 illustrates the structure of NRMSProp model and its layers with 50 epochs for training, where the values of the hyper parameters are: η is the learning rate (the default is 10-3), β1 and β2 are the smoothing parameters (β1 = 0.9, and β2 = 0.999), and ϵ is a small number and tis usually set as 10^−7^.

**Table 1:** The structure of the proposed CNN Model.

CNN Model		
Layer (type)	Output shape	Param
conv2d14 (conv2d)	(None, 26, 26, 32)	320
(maxpooling)	(None, 13, 13, 32)	0
flatten18 (flatten)	(None, 5408)	0
dense37 (dense)	(None, 128)	692,352
dense38 (dense)	(None, 10)	1290
Total params: 693,962 Trainable params: 693,962 Non-trainable params:		

Residual Network (ResNet)^42^ is a group of DNN models that has attained outstanding results on a variety of tasks associated with computer vision, including segmentation based on semantics, recognizing objects, and classification of images. The introduction of residual links, which enables the network to develop residual mappings that may be quickly optimized using gradient-based approaches, is the primary improvement of the ResNet models. The difference between the input and output of a group of convolutional layers, which is then added back to the input, is used for computing these residual mappings. Instead of trying to learn the complete mapping from scratching the network can learn to concentrate on the disparities between the input and the desired output in this manner. There are many depths of ResNet models, ranging from the original ResNet-18 to the far deeper ResNet-152. These models have already been trained using massive datasets like ImageNet. Here, ResNet V2 model is used to perform the experiments on NRMSporop. Figure 7 illustrates the structure of ResNet_V2 layers. The results of the two models on the three datasets are illustrated in the next section.

**Figure 7:**
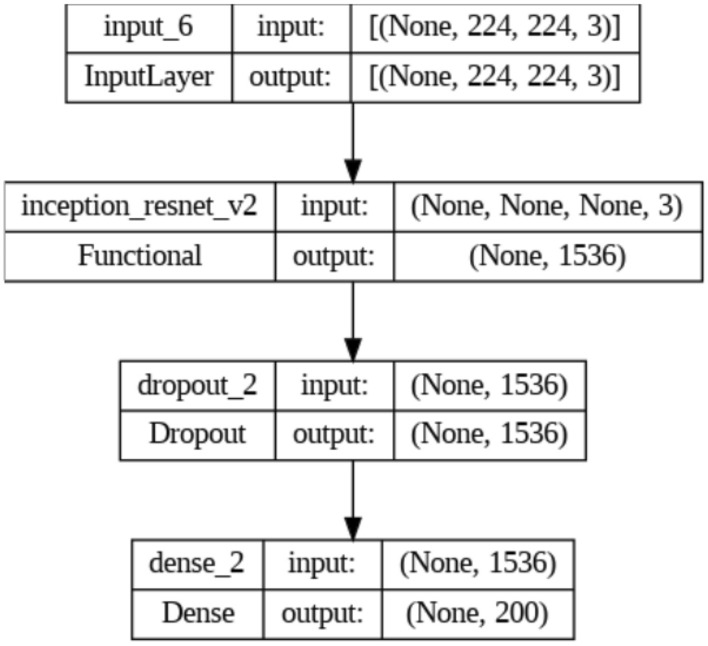
ResNet_V2 layers.

### Results of the experiments

Table 2 illustrates the overall performance of Adam on Fashion-MNIST dataset in which it achieves precision 98% which belongs to Class Trouser and Sandal, where 97% recall is the highest rate belongs to Class Sneaker and Bag. On the other hand, achieving 97% F1-score is the highest value belongs to Class Trouser. We present the classes of Fashion-MNIST dataset as (Class 1 refer to T-shirt/top, Class 2 refer to Trouser, Class 3 refer to Pullover, Class 4 refer to Dress, Class 5 refer to Coat, Class 6 refer to Sandal, Class 7 refer to Shirt, Class 8 refer to Sneaker, Class 9 refer to Bag and Class 10 refer to Ankle boot).

**Table 2:** The Precision, Recall and F1-score of Adam.

Fashion-MNIST dataset				
Classes	Precision	Recall	F1-score	Support
Class 1	0.83	0.78	0.80	1000
Class 2	0.98	0.96	0.97	1000
Class 3	0.81	0.80	0.80	1000
Class 4	0.84	0.87	0.86	1000
Class 5	0.71	0.88	0.78	1000
Class 6	0.98	0.93	0.95	1000
Class 7	0.71	0.56	0.63	1000
Class 8	0.91	0.97	0.94	1000
Class 9	0.94	0.97	0.96	1000
Class 10	0.95	0.94	0.95	1000
Accuracy			0.87	10,000
Macro avg	0.87	0.87	0.86	10,000
Weighted avg	0.87	0.87	0.86	10,000

Table 3 illustrates the overall performance of RMSPRop on Fashion-MNIST dataset in which it achieves precision 99% which belongs to Class Sandal, where 97% recall is the highest rate belongs to Class Trouser. On the other hand, achieving 98% F1-score is the highest value belongs to Class Trouser.

**Table 3:** The Precision, Recall and F1-score of RMSPRop.

Fashion-MNIST dataset				
Class	Precision	Recall	F1-score	Support
Class 1	0.74	0.88	0.80	1000
Class 2	0.98	0.97	0.98	1000
Class 3	0.84	0.69	0.76	1000
Class 4	0.86	0.88	0.87	1000
Class 5	0.80	0.72	0.76	1000
Class 6	0.99	0.93	0.95	1000
Class 7	0.58	0.62	0.60	1000
Class 8	0.91	0.96	0.93	1000
Class 9	0.98	0.95	0.96	1000
Class 10	0.94	0.96	0.95	1000
Accuracy			0.86	10,000
Macro avg	0.86	0.86	0.86	10,000
Weighted avg	0.86	0.86	0.86	10,000

Table 4 illustrates the overall performance of NRMSProp on Fashion-MNIST dataset in which it achieves precision 99% which belongs to Class Sandal and Trouser, where 98% recall is the highest rate belongs to Class Trouser, Sandal and Bag. On the other hand, achieving 98% F1-score is the highest value belongs to Class Trouser and Sneaker.

**Table 4:** The Precision, Recall and F1-score of NRMSProp.

Fashion-MNIST dataset				
Class	Precision	Recall	F1-score	Support
Class 1	0.81	0.88	0.85	1000
Class 2	0.99	0.98	0.98	1000
Class 3	0.78	0.86	0.82	1000
Class 4	0.86	0.91	0.89	1000
Class 5	0.83	0.80	0.82	1000
Class 6	0.99	0.98	0.98	1000
Class 7	0.80	0.63	0.71	1000
Class 8	0.95	0.98	0.98	1000
Class 9	0.96	0.98	0.97	1000
Class 10	0.98	0.95	0.97	1000
Accuracy			0.90	10,000
Macro avg	0.90	0.90	0.98	10,000
Weighted avg	0.90	0.90	0.98	10,000

Next experiment we present classes of CIFAR-10 dataset as (Class 1 refer to Airplane, Class 2 refer to automobile, Class 3 refer to Bird, Class 4 refer to Cat, Class 5 refer to deer, Class 6 refer to Dog, Class 7 refer to Frog, Class 8 refer to horse, Class 9 refer to Ship and Class 10 refer to truck). Table 5 illustrates the overall performance of Adam on CIFAR-10 dataset in which it achieves precision 83% which belongs to Class ship, where 81% recall is the highest rate belongs to Class truck. On the other hand F1-score is 78% is the highest value belongs to Class automobile.

**Table 5:** The Precision, Recall and F1-score of Adam.

CIFAR-10 dataset				
Class	Precision	Recall	F1-score	Support
Class 1	0.68	0.74	0.71	1000
Class 2	0.81	0.75	0.78	1000
Class 3	0.65	0.46	0.54	1000
Class 4	0.45	0.56	0.50	1000
Class 5	0.57	0.66	0.61	1000
Class 6	0.59	0.56	0.58	1000
Class 7	0.77	0.72	0.75	1000
Class 8	0.76	0.70	0.73	1000
Class 9	0.83	0.71	0.77	1000
Class 10	0.67	0.81	0.73	1000
Accuracy			0.67	10,000
Macro avg	0.68	0.67	0.67	10,000
Weighted avg	0.68	0.67	0.67	10,000

Table 6 illustrates the overall performance of RMSProp on CIFAR-10 dataset in which it achieves precision 91% which belongs to Class automobile, where 89% recall is the highest rate belongs to Class ship. On the other hand F1-score is 81% is the highest value belongs to Class automobile.

**Table 6:** The Precision, Recall and F1-score of RMSProp.

CIFAR-10 dataset				
Class	Precision	Recall	F1-score	Support
Class 1	0.69	0.69	0.69	1000
Class 2	0.91	0.73	0.81	1000
Class 3	0.73	0.49	0.58	1000
Class 4	0.52	0.43	0.62	1000
Class 5	0.73	0.53	0.62	1000
Class 6	0.63	0.60	0.62	1000
Class 7	0.81	0.72	0.76	1000
Class 8	0.57	0.85	0.69	1000
Class 9	0.63	0.89	0.74	1000
Truck	0.69	0.85	0.76	1000
Accuracy			0.68	10,000
Macro avg	0.69	0.68	0.67	10,000
Weighted avg	0.69	0.67	0.67	10,000

Table 7 illustrates the overall performance of NRMSPprop on CIFAR-10 dataset in which it achieves precision 85% which belongs to Class automobile, where 87% recall is the highest rate belongs to Class ship. On the other hand F1-score is 81% is the highest value belongs to Class automobile.

**Table 7:** The Precision, Recall and F1-score of NRMSprop.

CIFAR-10 dataset				
Class	Precision	Recall	F1-score	Support
Class 1	0.72	0.74	0.73	1000
Class 2	0.85	0.77	0.81	1000
Class 3	0.57	0.63	0.60	1000
Class 4	0.48	0.58	0.53	1000
Class 5	0.74	0.51	0.61	1000
Class 6	0.71	0.49	0.58	1000
Class 7	0.73	0.79	0.76	1000
Class 8	0.71	0.77	0.74	1000
Class 9	0.69	0.87	0.77	1000
Class 10	0.79	0.73	0.76	1000
Accuracy			0.69	10,000
Macro avg	0.70	0.69	0.69	10,000
Weighted avg	0.70	0.69	0.69	10,000

From Figs. 2, 3, 4, 5, 6, 7, the overall performance of NRMSProp is showed to be higher than Adam and Rmsprop in reaching high value in most classes of Fashion-MNIST and CIFAR-10 datasets.

The second criteria for the evaluation stage is constructing confusion matrix^43^, in which it gives an accurate assessment of the model's accuracy in terms of true positives, true negatives, false positives, and false negatives. This aids in comprehending the model's performance and locating potential improvement areas. Also, it offers a more in-depth and a picture of the model's performance. It can assist in pinpointing certain instances where the model is functioning successfully or incorrectly. The model's general effectiveness can be improved by detecting problem areas with the use of the confusion matrix, which can also be used to guide parameter adjustment and refining. Three confusion matrices are constructed for each dataset as given below.

**Figure 8:**
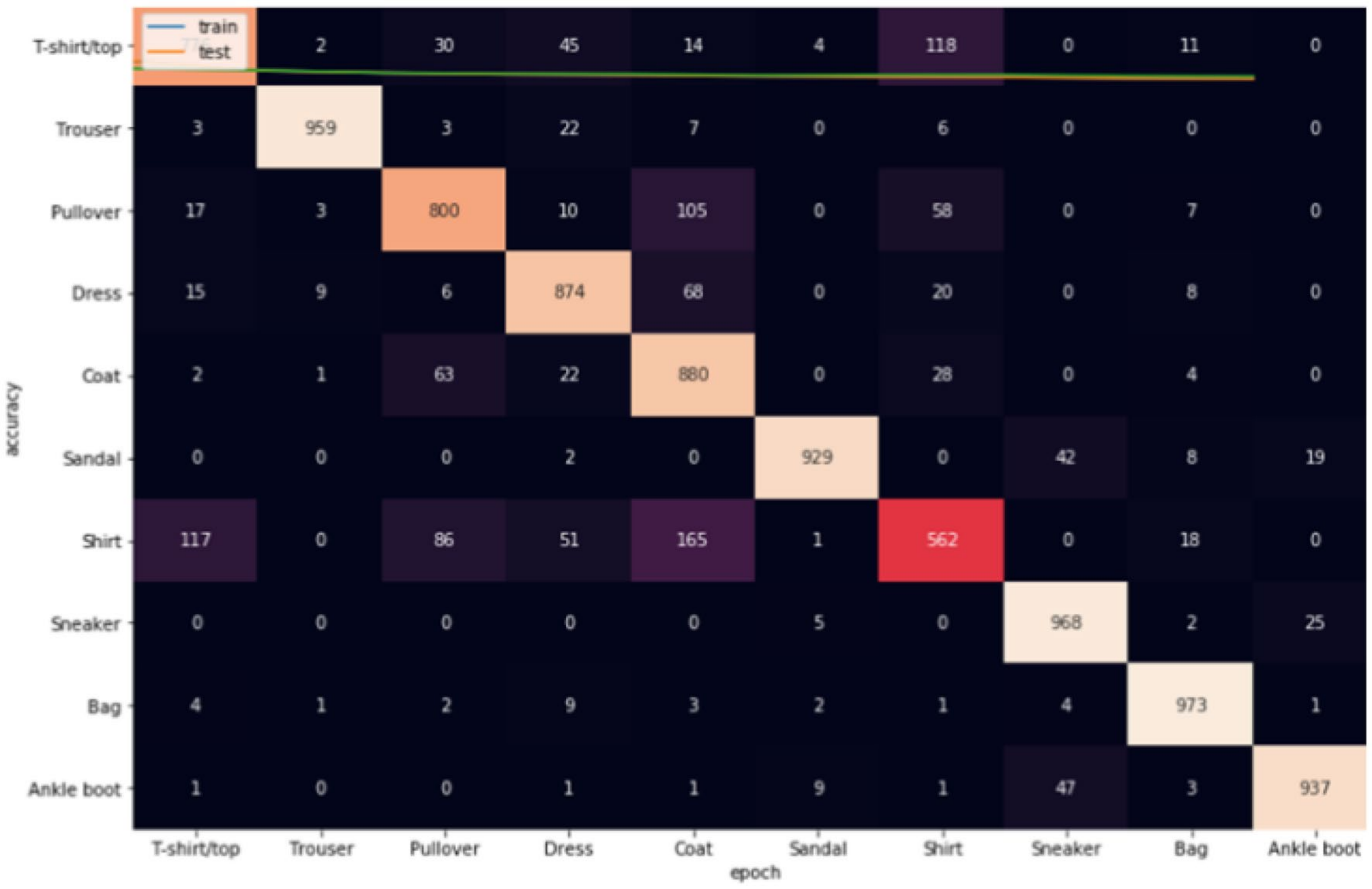
Confusion matrix of Adam for Fashion-MNIST dataset.

**Figure 9:**
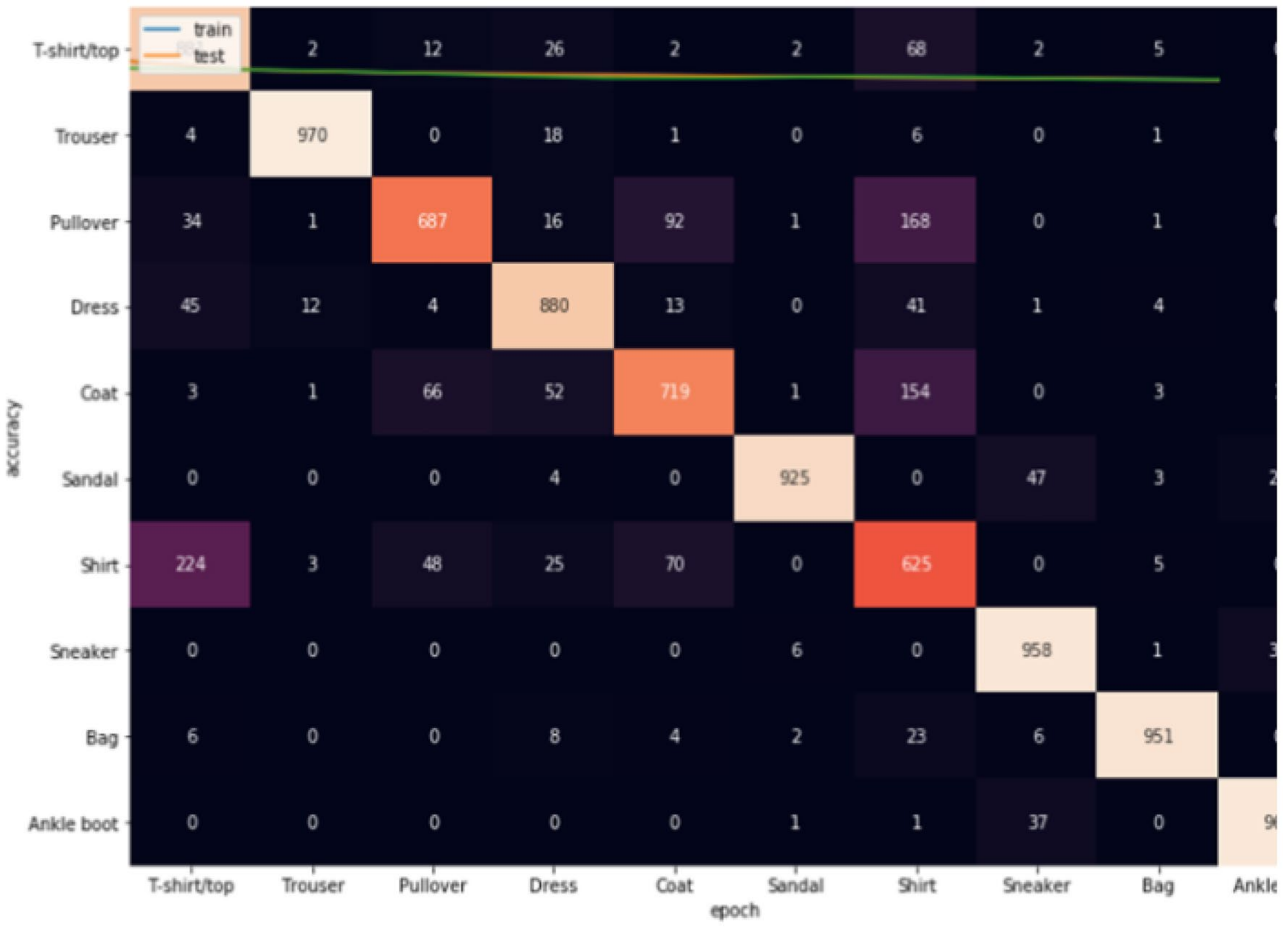
Confusion matrix of Rmsprop for Fashion-MNIST dataset.

**Figure 10:**
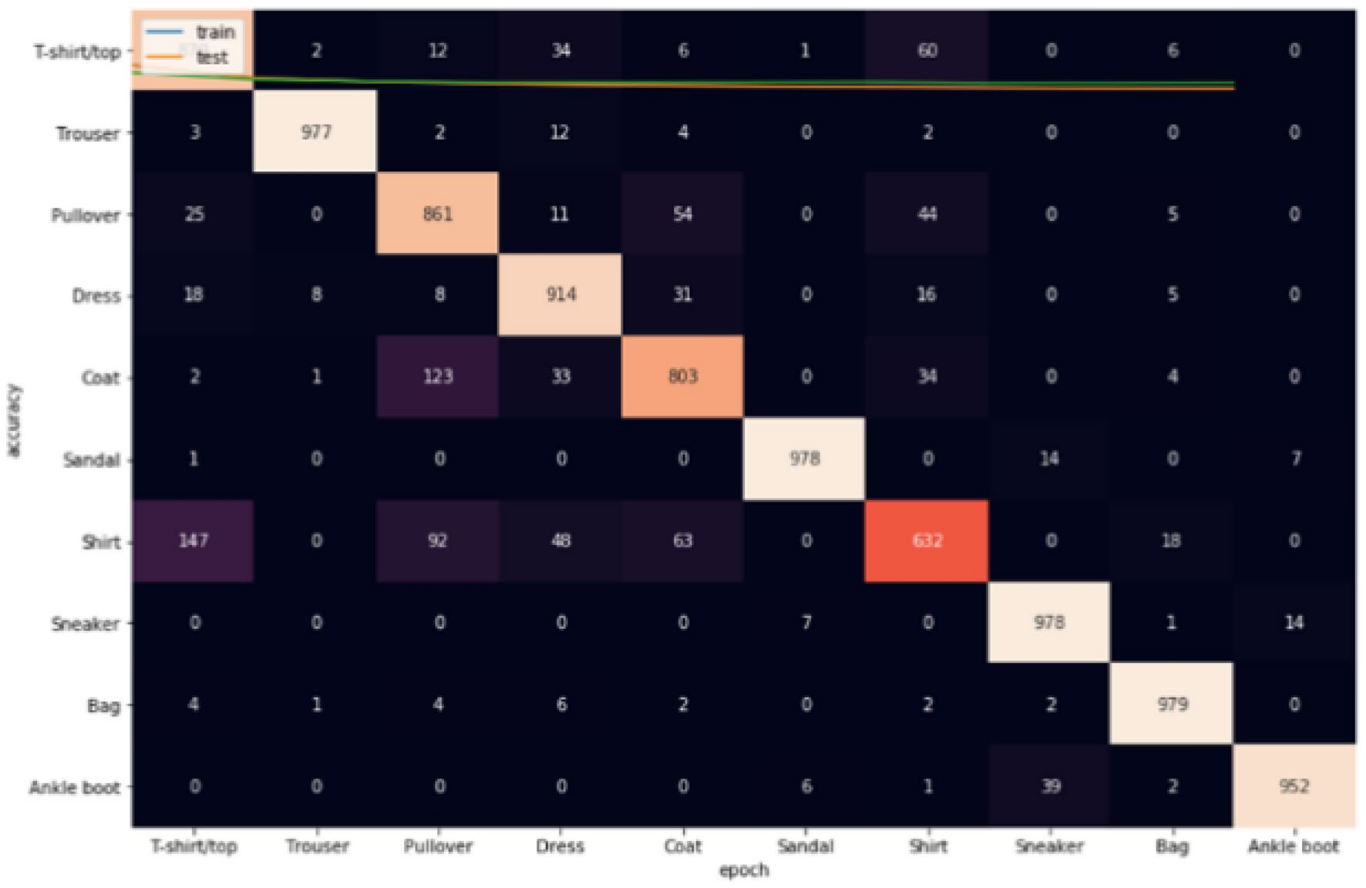
Confusion matrix of NRMSprop for Fashion-MNIST dataset.

Figures 8, 9, 10 show the confusion matrices for Fashion-MNIST datasets. Figure 8 illustrates that Adam is getting confused mostly between similar classes in comparison between actual and predicted values. Some of the higher values of negative classification are between these classes: (T-shirt/top and Shirt) (Pullover and Shirt) (Coat and Shirt) (Coat and Pullover) with values (117, 68, 165, 105). Figure 9 illustrates that RMSProp is getting confused in many classes in comparison between actual and predicted values. Some of the higher values of negative classification are between these classes: (T-shirt/top and Shirt) (Pullover and Coat) (Dress and Coat) (Coat and Pullover) (Shirt and coat) (Shirt and Pullover) with values (224, 66, 52, 92, 154, 168). Figure 10 illustrates NRMSprop is getting confused just in very similar classes in comparison between actual and predicted values. Some of the higher values of negative classification are between these classes: (T-shirt/top and Shirt) (Pullover and Coat) (Pullover and Shirt) with values (147, 123,92). Depending on these experiments on Fashion-MNIST dataset, NRMSprop is shown to be the less confusion degree than Adam and Rmsprop.

**Figure 11:**
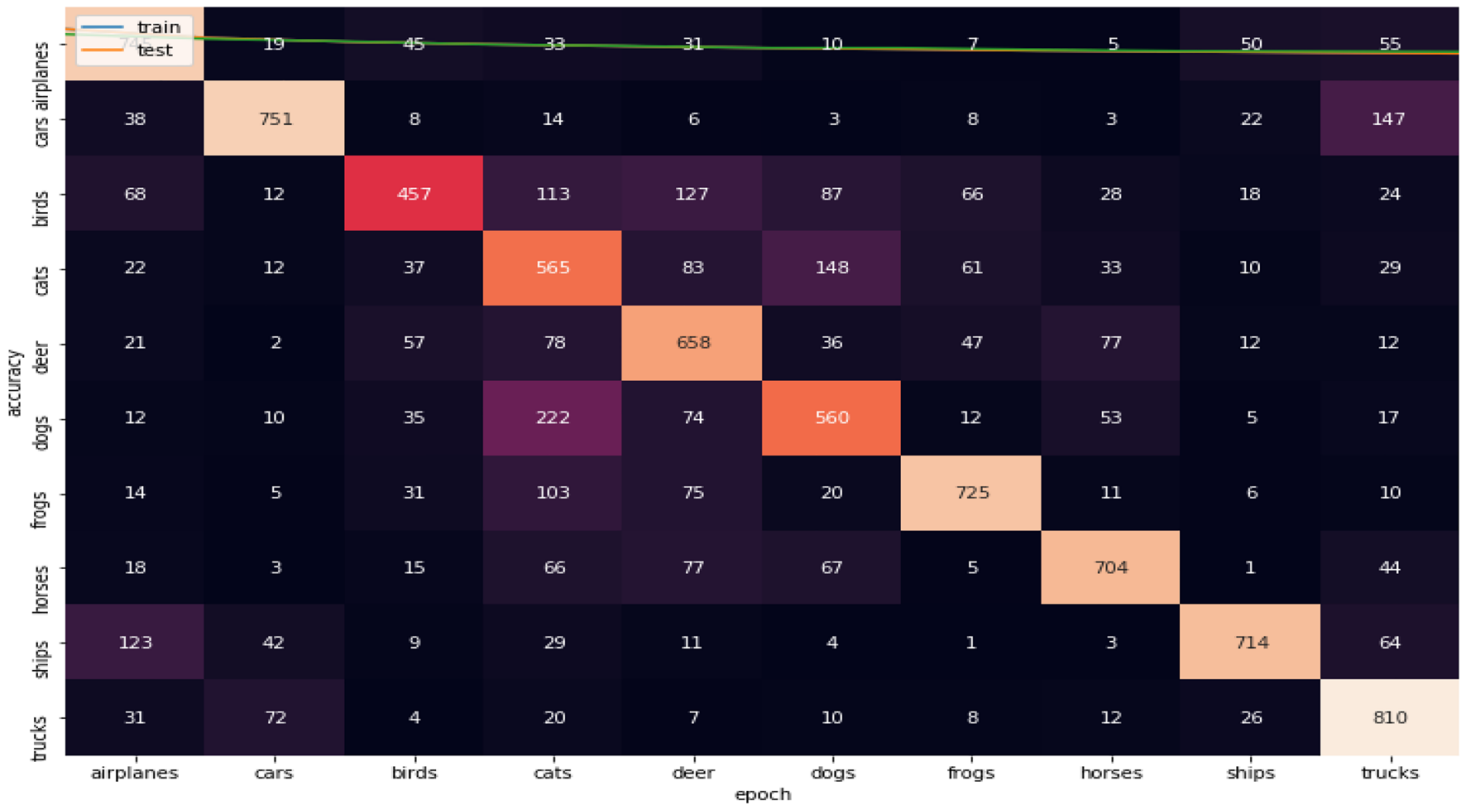
Confusion matrix of Adam for CIFAR-10.

**Figure 12:**
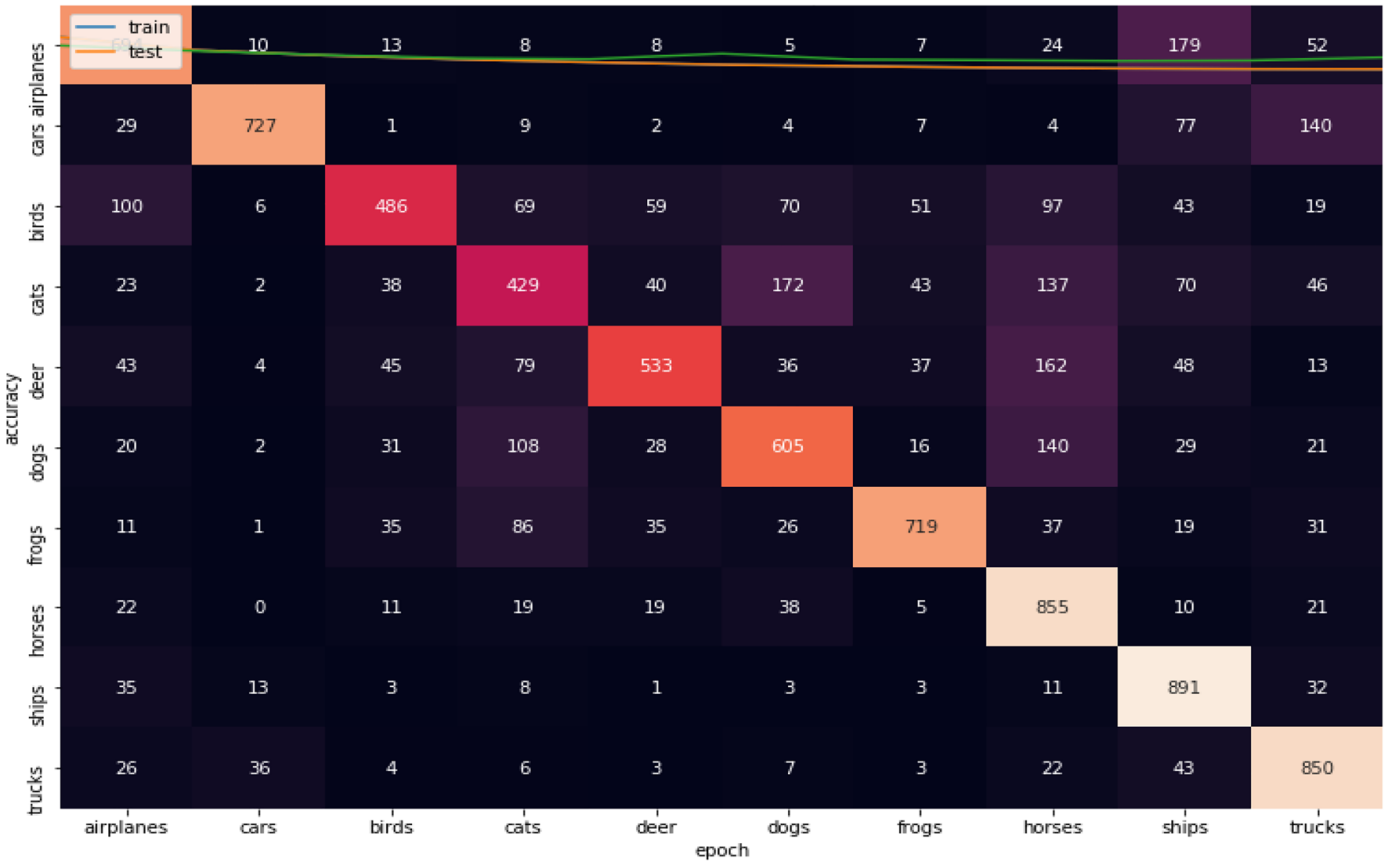
Confusion matrix of Rmsprop for CIFAR-10.

**Figure 13:**
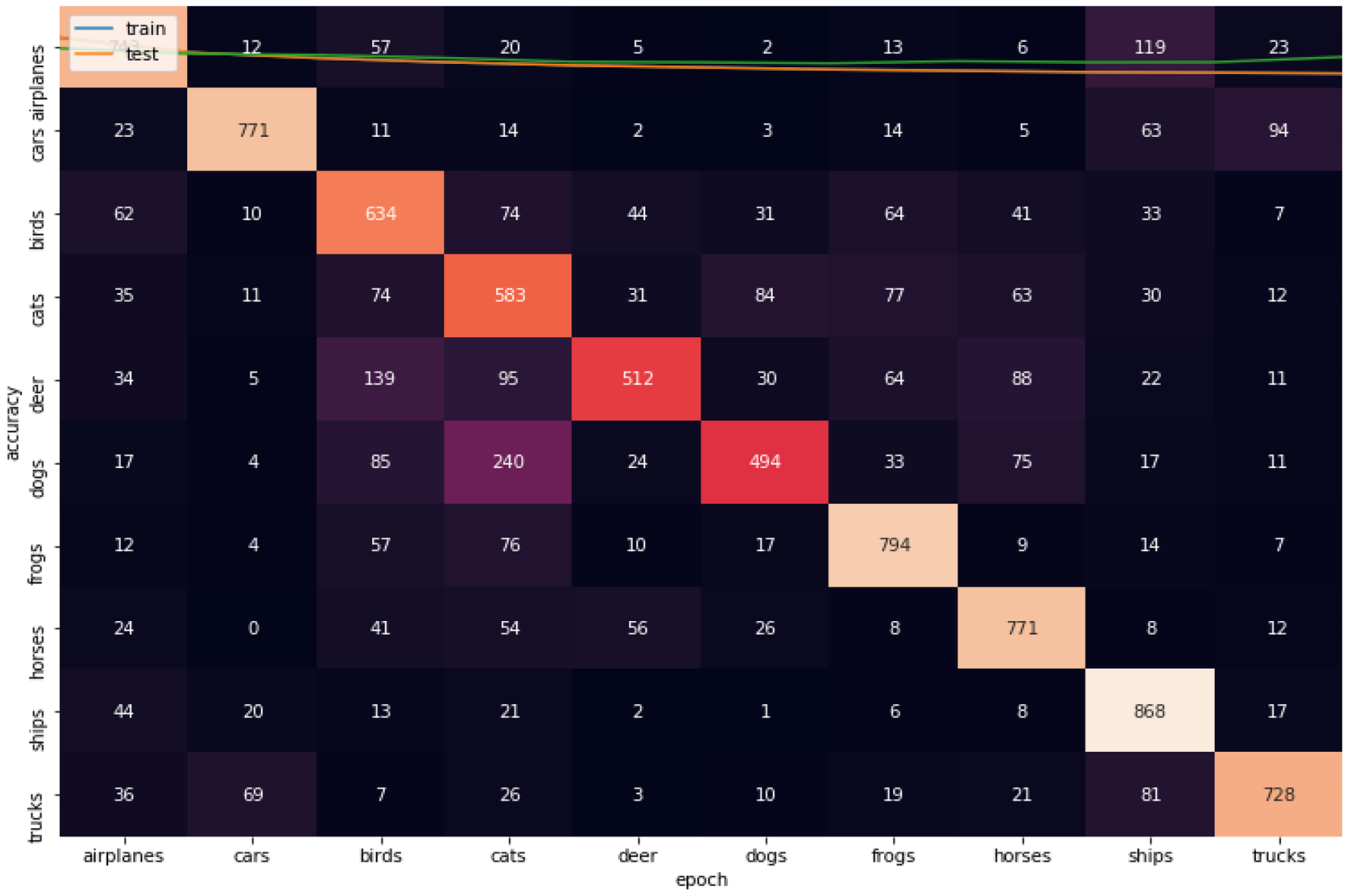
Confusion matrix of NRMSprop or CIFAR-10.

Figures 11, 12, 13 show the confusion matrices for CIFAR-10 datasets. Figure 11 illustrates that Adam is getting confused in many classes in comparison between actual and predicted values. By focus on some of higher values of negative classification are between these classes we found many classes have high confusion value like (airplanes, cats, deer, dogs, and trucks). Figure 12 illustrates that that RMSProp is getting confused in many classes in comparison between actual and predicted values. By analyzing the behavior or RMSProp, the most of values are shown to be very close to each other and RMSProp is hardly distinguished images between overall classes in dataset. Figure 13 illustrates that NRMSProp is getting confused in less number of classes in comparison between actual and predicted values compared with Adam and RMSProp. NRMSProp. By focus on some of higher values of negative classification are between these classes we found that classes with have high confusion values are (birds, cats, ships). Depending on these experiments on CIFAR-10 dataset, NRMSprop is shown to be the less confusion degree than Adam and RMSProp.

**Figure 14:**
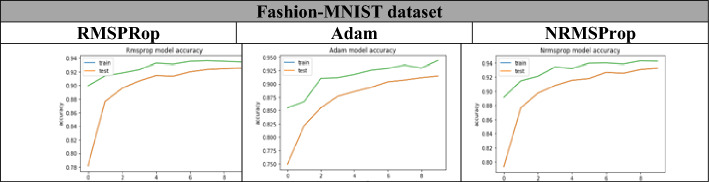
The accuracy of training and test curves of RMSPRop, Adam, and NRMSProp on Fashion-MNIST dataset.

**Figure 15:**
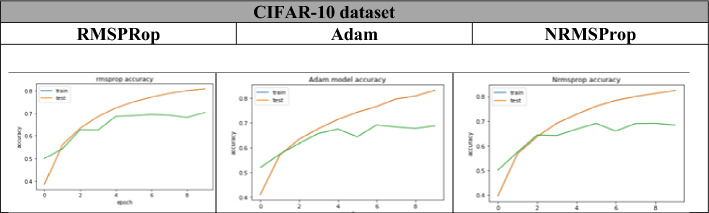
The accuracy of training and test curves of RMSPRop, Adam, and NRMSProp on CIFAR-10 dataset.

**Figure 16:**
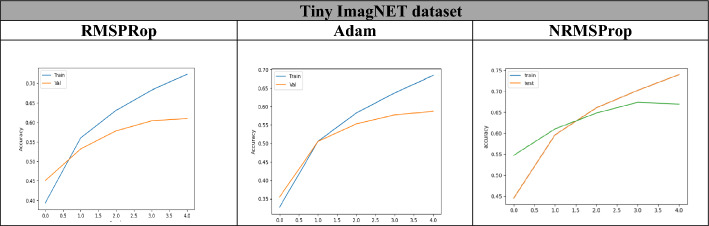
The accuracy of training and test curves of RMSPRop, Adam, and NRMSProp on Tiny ImagNET dataset.

From Table 8, Figs. 14, 15, 16, it is clear that the proposed NRMSProp achieves good results in comparison with the other optimizers under all measurement criteria. The overall performance of the NRMSProp optimizer is more efficient after adding the Nestrove term to its steps. Therefore, the power of Nestrove can be utilized in to enhance the accuracy and to speed up the process of the optimizer in general. Moreover, NRMSProp has an effect feature that keeps the history of the current point to be more efficient in speeding up the process of taking the decision of which direction should be chosen. When adding Nestrove to NRMSProp’s steps, it gives NRMSProp the power to take a big step in the right direction by look ahead for projected positions not only the actual position and by adapting the learning step for each iteration depend on the minima position.

**Table 8:** The accuracy results of Adam, RMSProp, and NRMSProp.

Dataset	Adam	RMSProp	NRMSProp
Fashion-MNIST	0.91	0.86	0.97
CIFAR-10	0.71	0.82	0.84
Tiny ImagNET	0.69	0.70	0.72

## Conclusion

This paper proposed NRMSProp as a modified version of the Adaptive optimizer to increase its efficiency. It takes RMSProp algorithm one step further since the efficiency of the Nestrove technique is combined with RMSProp optimizer. Moreover, to examine the proposed algorithm NRMSProp, experiments are conducted on the Adam, RMSProp, and NRMSProp optimizers on Fashion-MNIST, CIFAR-10 and Tiny-ImagNet datasets. Accuracy, Precision, Recall, F1-score, and confusion matrix are used to evaluate NRMSProp and compare its performance with Adam and RMSProp optimizers on different datasets. The results showed that the NRMSProp achieved high accuracy udder all measurements with fast convergence without noticeably increasing complexity.

## Data Availability

The datasets (Fashion-MNIST, cifar and ImagNet) used during the current study available in the following links respectively: Xiao et al.^33^. Available: arXiv:1708.07747, https://www.cs.toronto.edu/~kriz/cifar.html, https://tiny-imagenet.herokuapp.com.
